# Identification and validation of a robust inflammation-related three-gene signature for the diagnosis of neonatal necrotizing enterocolitis via machine learning integration

**DOI:** 10.1007/s00383-026-06659-1

**Published:** 2026-09-26

**Authors:** Lihua Yuan, Xiaobo Wang, Kenneth Kak Yuen Wong

**Affiliations:** 1 Division of Pediatric Surgery, Department of Surgery, The University of Hong Kong Shenzhen Hospital, Shenzhen, China; 2 Division of Pediatric Surgery, Department of Surgery, Queen Mary Hospital, The University of Hong Kong, Pokfulam Road, Hong Kong SAR, China; 3 The Seventh Affiliated Hospital, Sun Yat-sen University, Shenzhen, 518107 Guangdong P.R. China

**Keywords:** Neonatal necrotizing enterocolitis, Machine learning, Diagnosis

## Abstract

**Purpose:**

Neonatal necrotizing enterocolitis (NEC) is a severe gastroesophageal emergency in neonates, especially premature infants, with acute inflammation and necrosis. Due to the nonspecific nature of early clinical manifestations, delayed diagnosis remains an issue often associated with high mortality and morbidity, hence finding molecular biomarkers with high diagnostic specificity is critical for better clinical outcomes. This work combines transcriptomic data with machine learning algorithms to identify a diagnostic signature that strongly correlates to NEC.

**Methods:**

The GSE64801 transcriptome dataset from GEO was trained and the independent GSE46619 dataset was validated externally. A bioinformatics pipeline was developed to detect differentially expressed genes (DEG) between NEC and control tissues intersected with 935 known inflammation-related genes (IRGs) to identify inflammation-specific candidates. For feature robustness, the combinatorial screening strategy involving least absolute shrinkage and selection operator regression and Boruta random forest algorithm was used. Diagnostic performance of identified core biomarkers was validated across cohorts. A diagnostic nomogram was constructed and performance was evaluated using Receiver Operating Characteristics (ROC) curves, calibration plots, Decision Curve Analysis (DCA). Various downstream analyses including GSEA, regulatory network construction, drug target prediction were performed.

**Results:**

In the training cohort 452 DEGs were identified, down to 20 candidates after intersecting with IRGs. The two machine learning strategies identified three key biomarkers: REG3A, CXCL9 and Kininogen 1 (KNG1). Validation assays showed that these genes are upregulated in NEC tissues in both cohorts. The nomogram constructed from this three genes was more diagnostically effective and achieved Area Under the Curve (AUC) of 0.956 in the training cohort. Mechanistically GSEA plays a significant role in key immune-inflammatory processes such as chemokine signaling, intestinal immune networks and graft-versus-host responses. Further upstream transcriptional regulation network was constructed and potential therapeutic agents such as avoralstat were predicted.

**Conclusion:**

We identified and validated a novel three gene inflammatory signature (REG3A, CXCL9, KNG1) high diagnostic accuracy for NEC. These biomarkers are involved in the basic pathophysiology of the disease, providing a scientific basis for early diagnostic tools and precise treatments.

## Introduction

Neonatal necrotizing enterocolitis (NEC) is a sever intestinal inflammation, ischemia and necrosis characterized by high levels of intestinal inflammation, ischemia and necrosis, and severely affecting newborns globally especially low birth weight[1]. The disease progresses rapidly, from mild feeding intolerance to intestinal perforation, peritonitis and potentially systemic septic shock. Survivalists suffer from severe short bowel syndrome, intestinal stenosis, growth retardation and long term neurodevelopmental disorders which burden families [2].

The pathophysiology of NEC is a complex combination of risk factors including immature intestinal barrier function, intestinal microbiome dysbiosis, ischemia-reperfusion injury, and excessive activation of the host immune system [3]. An uncontrolled inflammatory cascade is widely recognized as driving from initial injury to irreversible intestinal necrosis [4]. In the clinical diagnosis of NEC, most of the symptoms are nonspecific (i.e. abdominal distension, bloody stool) and advanced signs like pneumatosis intestinalis seen on abdominal X-rays. However, this approach often leads to delay in identifying NEC, and often children miss the critical opportunity for treatment/u. Hence, biomarkers such as molecular markers that mirror basic pathological processes play an important role in addressing the diagnostic and therapeutic challenges associated with NEC. Biomarkers, especially molecular markers, are important tools for early and precise diagnosis since they provide valuable insights into the pathology of NEC. Identifying such markers allows healthcare professionals to improve diagnostic accuracy and tailor treatment approach accordingly [5, 6].

The high-throughput sequencing technology and the bioinformatics tools allow us to sequence disease molecular profiles from whole genome level. Specifically, we focus on inflammation related genes (IRGs) which are critical for NEC pathology. We aim at finding candidate markers from large datasets. Also, advanced machine learning techniques such as LASSO regression [7] and Boruta algorithm allow us to obtain core feature combinations with high predictive value from high-dimensional data, which can build diagnostic model of high performance and strong generalization.

We applied a systems biology approach. We searched public transcriptomic data to detect inflammation-associated DEGs in NEC, applied two different machine learning algorithms (LASSO and Boruta) and found a robust three-gene signature. We performed multidimensional bioinformatic analyses to study the biological functions, regulatory networks, and potential therapeutic targets of these biomarkers.

## Methods

### Data acquisition and preprocessing

The NEC transcriptomic dataset GSE64801 was obtained from GEO to train, and the independent dataset GSE46619 was used as a validation dataset to test the generalization of the model. Samples are provided in Supplementary Materials. 935 IRGs were selected from a published study (PMID: 36467500).

### Identification of differentially expressed inflammation-related genes

Raw count data from the training set were normalized and differential expression analysis performed using DESeq2 R package. P values were adjusted for multiple testing using Benjamini-Hochberg procedure. Genes with adjusted P value 0.05 and |log2 fold change (FC)| > 1 were characterized as differential expression genes. Intersection analysis between DEGs and IRG list was performed to identify inflammation specific candidate genes involved in NEC pathology.

### Functional enrichment and PPI network analysis

To illustrate functions of the biological processes, GO annotation and KEGG pathway enrichment were performed using clusterProfiler R package (P 0.05) and PPI network using STRING database (v11.5) with medium confidence score (> 0.4) to visualize functional synergies among candidate proteins.

### Machine learning-based feature selection

We used two algorithms to ensure robust selection of biomarkers. First we constructed the LASSO logistic regression model using glmnet R package, L1 regularization penalty shrinking redundant variables to zero and selecting features at the best lambda value. Second we applied Boruta feature selection algorithm which compares original attributes to random “shadow” features to select all important features associated with result.

### Identification and validation of core biomarkers

Genes identified by both LASSO and Boruta algorithms were designated as core biomarkers. Their expression patterns were systematically validated in both the training and external validation cohorts using the Wilcoxon rank-sum test. A P-value < 0.05 was considered statistically significant.

### Construction and evaluation of the diagnostic nomogram

A diagnostic nodeogram consisting of the core biomarkers was developed to predict individual NEC risk. Model performance was assessed in three dimensions: (1) Discrimination based on the Area Under the ROC Curve (AUC) in pROC package; (2) Calibration based on calibration curves plotted and Hosmer-Lemeshow goodness-of-fit test; (3) Clinical utility based on Decision Curve Analysis (DCA) in rmda package to estimate net benefits at different threshold probabilities.

### Multi-omics and regulatory network analysis

In order to understand biological significance of biomarkers we performed following analyses: (1) Chromosome mapping: Visualized using RCircos package; (2) Interaction Network: Co-expression and physical interactions explored using GeneMANIA database; (3) Pathway Activity: Gene Set Enrichment Analysis (GSEA) was carried out for pathways associated with biomarker expression; (4) Transcriptional Regulation: Key transcription factors (TF) predicted using JASPAR database using NetworkAnalytics platform to build TF-mRNA regulatory network; (5) Drug Prediction: Potential therapeutic agents were checked using Drug Gene Interaction Database (DGIdb).

### Statistical analysis

All statistical analyses were performed using R software (version 4.2.1). Continuous variables were compared using the Wilcoxon rank-sum test. Statistical significance was defined as two-sided *P* < 0.05 unless otherwise specified.

## Results

### Identification of inflammation-related candidate genes in NEC

**Fig. 1:**
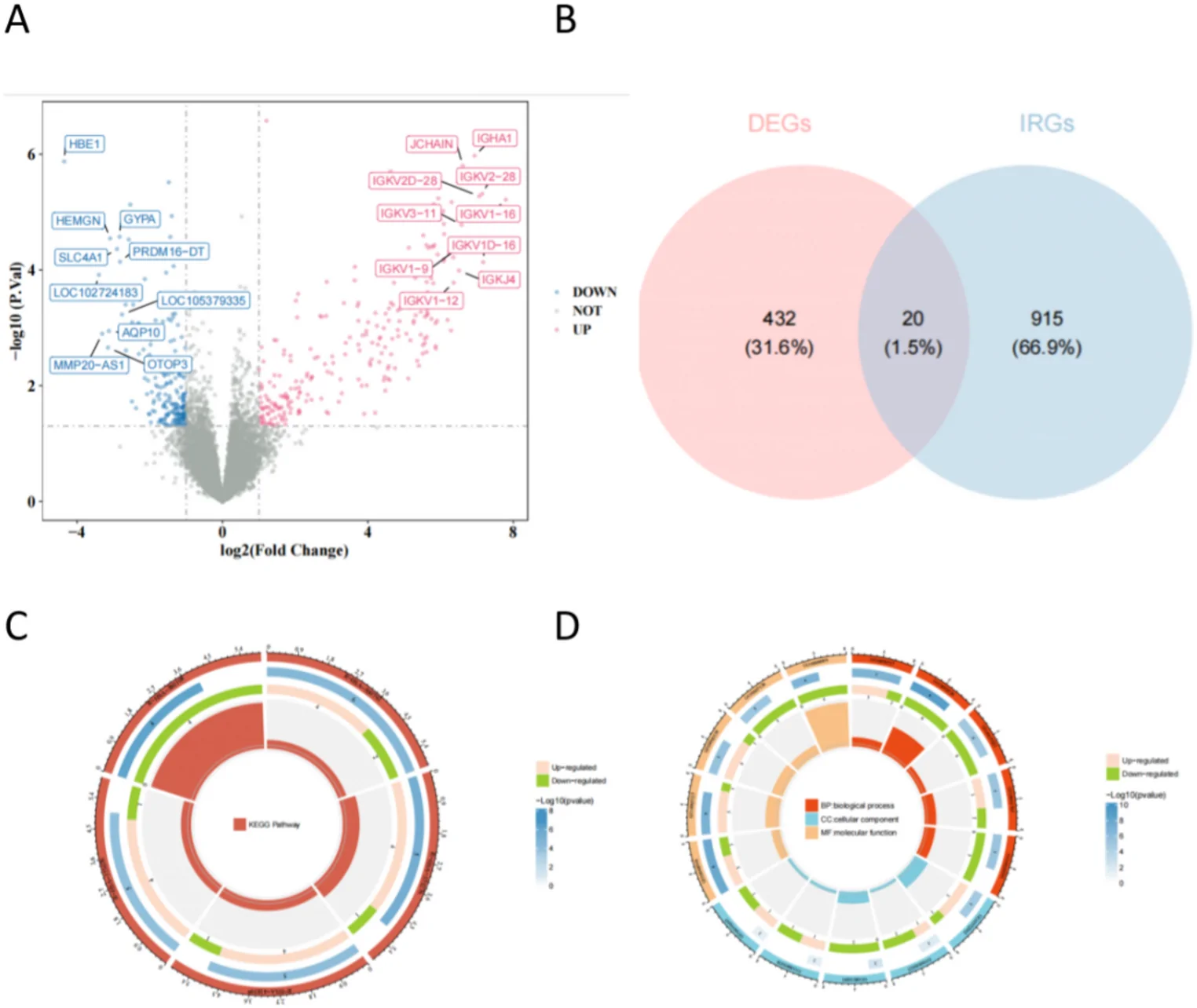
Identification of inflammation-related DEGs in NEC. **A** Volcano plot of DEGs in GSE64801 (*P* < 0.05, |log2FC| > 1). Red: upregulated; Blue: downregulated. **B** Venn diagram showing 20 candidate genes overlapping between DEGs and IRGs. **C–D** Circular plots for GO (C) and KEGG (D) enrichment analyses of the 20 candidate genes

To investigate the molecular alterations associated with inflammation in NEC, we performed differential expression analysis on the GSE64801 training dataset. A total of 452 DEGs were identified between NEC and control tissues, including 244 upregulated and 208 downregulated genes, as visualized in the volcano plot (Fig. 1A). To focus specifically on inflammatory mechanisms, we intersected these DEGs with a predefined list of 935 IRGs, resulting in 20 candidate inflammation-related genes (Fig. 1B). Functional enrichment analysis was conducted to elucidate the biological roles of these 20 candidates. The circular visualization of GO analysis revealed significant enrichment in biological processes such as “cytokine activity” and “chemokine receptor binding” (Fig. 1C). Similarly, KEGG pathway analysis highlighted the “Cytokine-cytokine receptor interaction” and “Chemokine signaling pathway” as the most prominent pathways, suggesting a robust immune-driven pathology (Fig. 1D).

### Screening of robust diagnostic biomarkers via dual machine learning

**Fig. 2:**
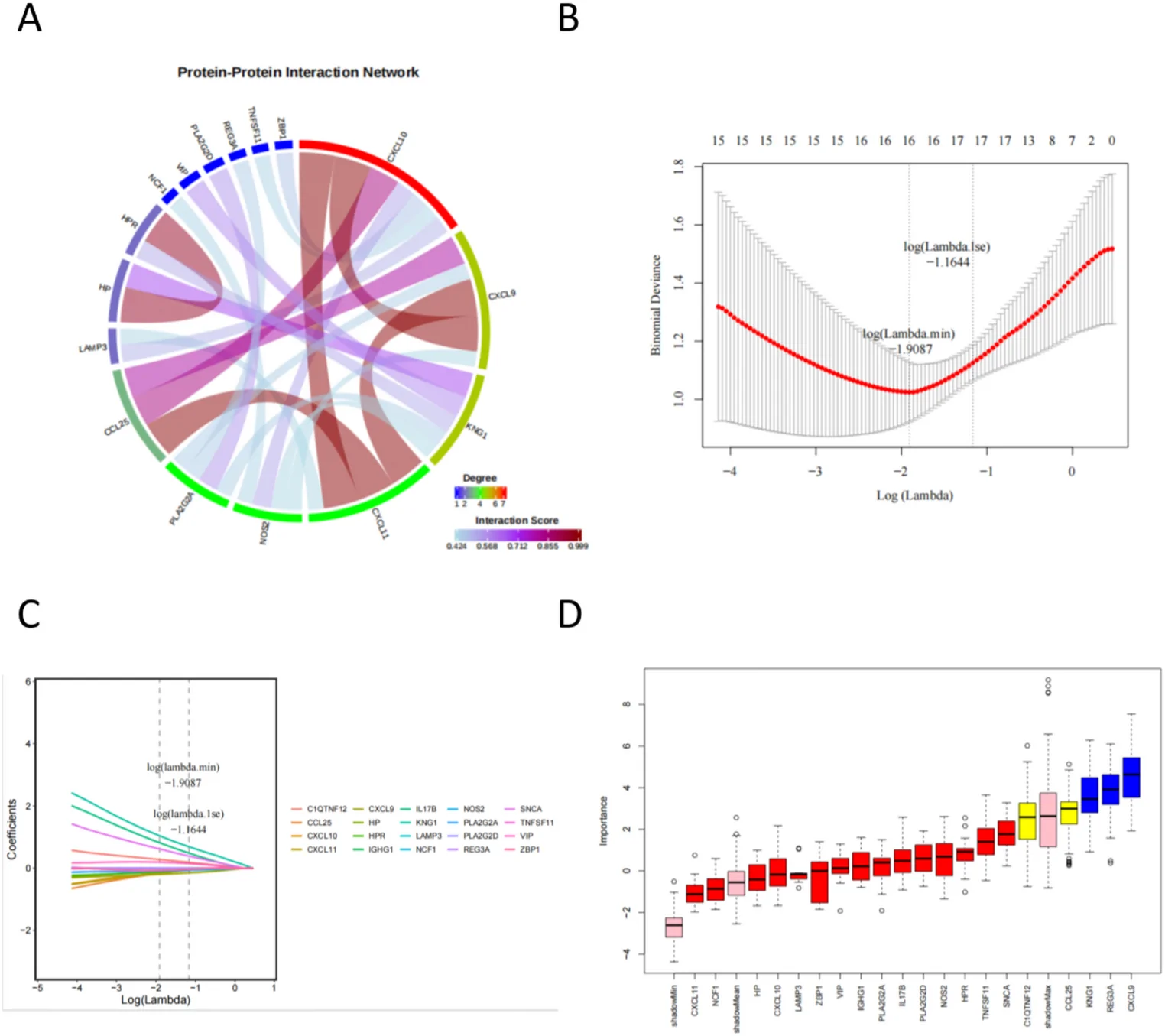
Screening of diagnostic biomarkers via machine learning. **A** PPI network of the 20 candidate genes. **B** LASSO coefficient profiles. **C **Cross-validation for LASSO tuning parameter selection. **D** Boruta feature selection (Blue: confirmed important features). **E** Venn diagram identifying three core biomarkers (REG3A, CXCL9, KNG1) shared by LASSO and Boruta algorithms

**Fig. 3:**
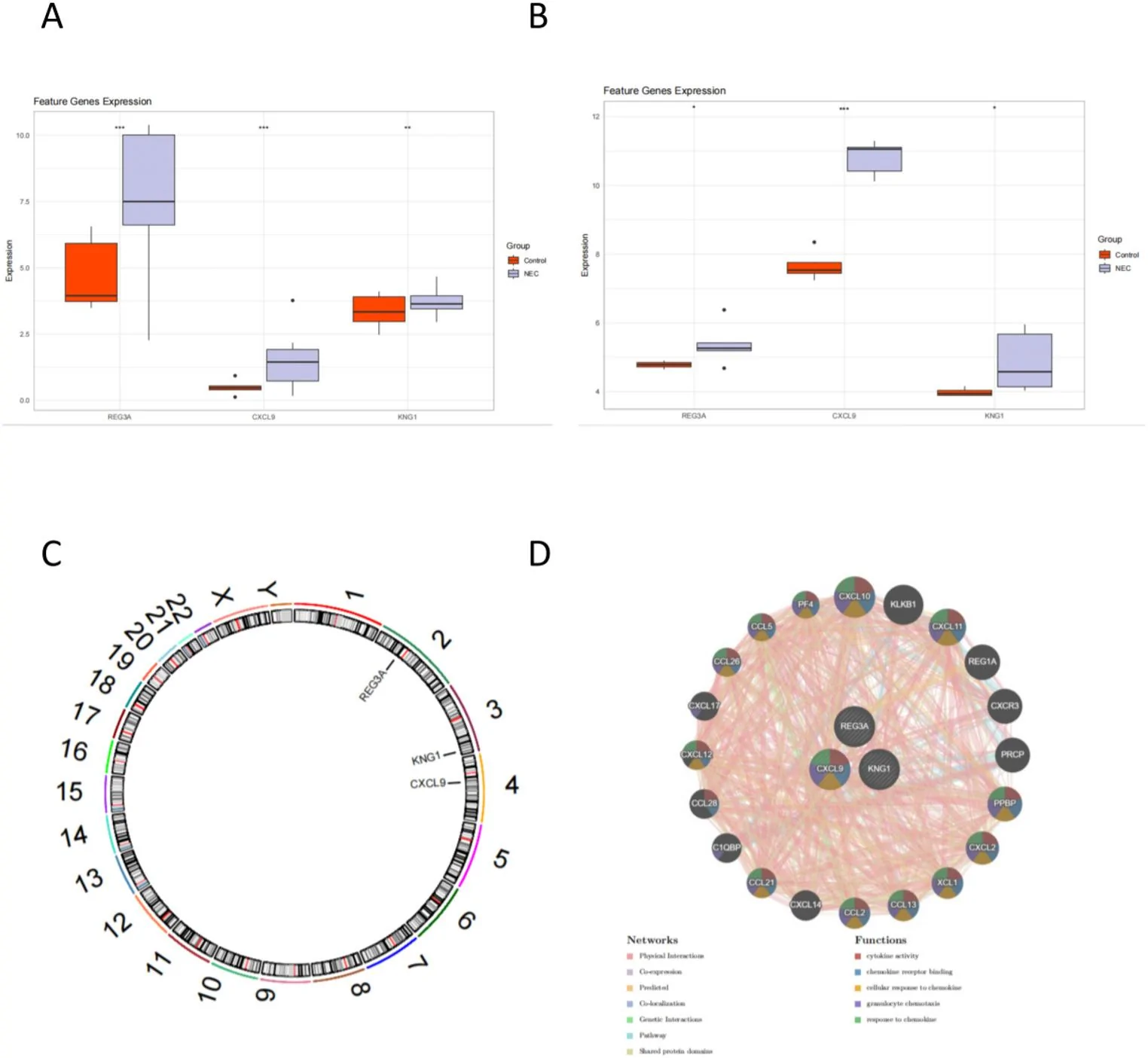
Validation and interaction of the three biomarkers. **A–B** Expression levels of REG3A, CXCL9, and KNG1 in the training set GSE64801 **A** and validation set GSE46619 **B** (Purple: NEC; Red/Orange: Control). **C** Chromosomal locations of the biomarkers. **D** GeneMANIA network showing interactions between biomarkers and related genes. (*P* < 0.05, P* < 0.01, *P* < 0.001)

We first constructed a Protein-Protein Interaction (PPI) network, which demonstrated dense functional connections among the 20 candidate proteins, indicating a coordinated inflammatory module (Fig. 2A). To identify the most robust diagnostic features, we employed a dual machine-learning strategy. The LASSO regression analysis minimized overfitting and identified 17 potential features with non-zero coefficients at the optimal lambda (3.3 Validation of Expression Patterns and Multi-Omics Characterization The expression levels of the three core biomarkers were validated in both the training (GSE64801) and independent validation (GSE46619) cohorts. Consistent with our hypothesis, REG3A, CXCL9, and KNG1 were significantly upregulated in NEC tissues compared to controls in both datasets (*P* < 0.05 to *P* < 0.001), demonstrating excellent biological reproducibility (Fig. 3A-B). Chromosomal mapping revealed that these genes are located on distinct chromosomes: REG3A on chromosome 2, KNG1 on chromosome 3, and CXCL9 on chromosome 4 (Fig. 3C). Furthermore, GeneMANIA network analysis illustrated a complex interactome, showing that these biomarkers share co-expression patterns and physical interactions with 20 other immune-related genes, such as CXCL10 and CCL5 (Fig. 3D).

### Construction and clinical evaluation of the diagnostic nomogram

**Fig. 4:**
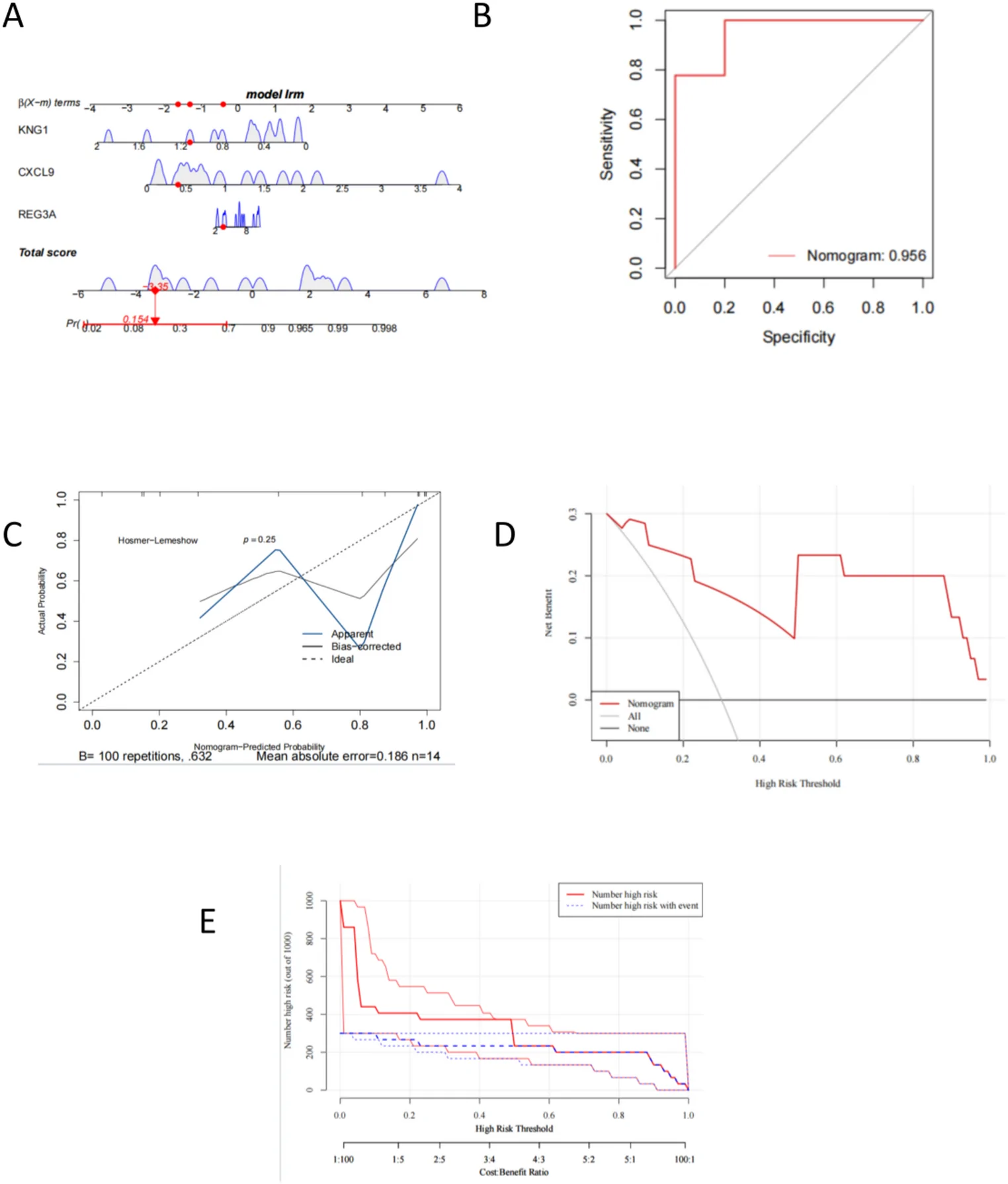
Construction and evaluation of the diagnostic nomogram. **A** Nomogram predicting NEC risk based on the three biomarkers. **B** ROC curve in the training set (AUC = 0.956). **C** Calibration curve assessing prediction accuracy (Ideal line: diagonal). **D** Decision Curve Analysis (DCA) showing net clinical benefit. **E** Clinical Impact Curve (CIC) evaluating clinical utility

Based on the three-gene signature, we constructed a diagnostic nomogram to predict the risk of NEC. In this model, the expression level of each gene corresponds to a specific score, and the total score indicates the probability of disease (Fig. 4A). The model exhibited superior discrimination, with an Area Under the Curve (AUC) of 0.956 in the training set (Fig. 4B). The calibration curve showed high consistency between the predicted and observed probabilities, indicated by the alignment with the ideal diagonal line (Fig. 4C). Moreover, Decision Curve Analysis (DCA) demonstrated that the nomogram provided a higher net benefit than “treat-all” or “treat-none” strategies across a wide range of threshold probabilities (Fig. 4D). The Clinical Impact Curve (CIC) further confirmed the model’s clinical utility, showing a high match between predicted high-risk cases and actual events (Fig. 4E).

### Underlying molecular mechanisms revealed by GSEA

**Fig. 5:**
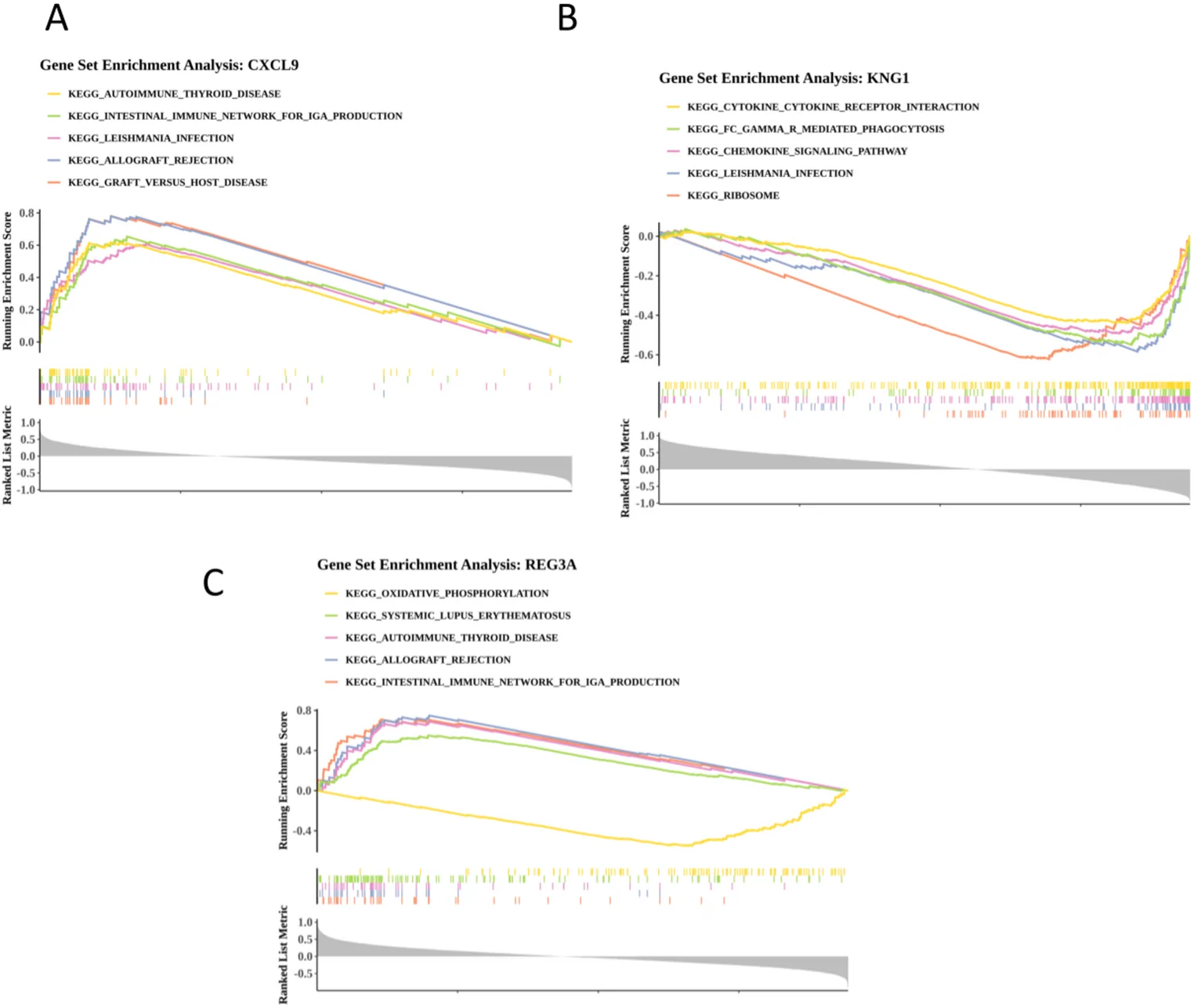
GSEA enrichment analysis. **A**–**C** GSEA plots showing key KEGG pathways enriched in samples with high expression of CXCL9 **A** KNG1 **B**, and REG3A **C**, highlighting immune and inflammatory pathways

To explore the biological pathways driven by these biomarkers, we performed single-gene GSEA. For CXCL9, high expression was positively correlated with “Autoimmune thyroid disease,” “Intestinal immune network for IgA production,” and “Allograft rejection,” reflecting an intense adaptive immune response (Fig. 5A). In the case of KNG1, enriched pathways included “Cytokine-cytokine receptor interaction” and “Chemokine signaling pathway,” linking vascular regulation to inflammatory signaling (Fig. 5B). Regarding REG3A, while it was positively correlated with immune pathways such as “Systemic lupus erythematosus,” its high expression was notably negatively correlated with “Oxidative phosphorylation” (indicated by the downward curve in yellow), suggesting a metabolic shift in the inflamed epithelium (Fig. 5C).

### Upstream regulatory networks and drug prediction

**Fig. 6:**
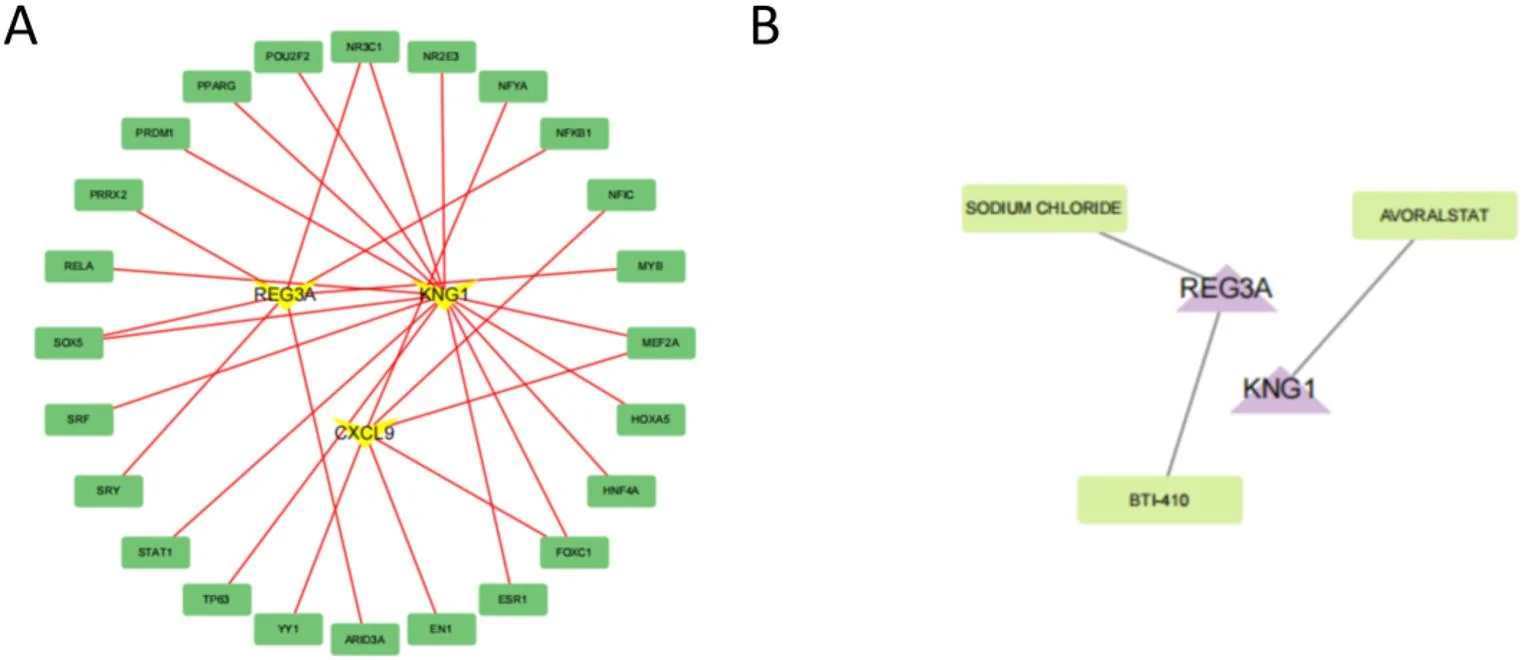
Regulatory network and drug prediction. **A** TF-mRNA regulatory network (Purple: biomarkers; Green: transcription factors). **B** Drug-Gene interaction network predicting potential therapeutic agents (Yellow: biomarkers; Pink: drugs).

Finally, we investigated the regulatory landscape and therapeutic potential of the biomarkers. The TF-mRNA regulatory network predicted key transcription factors, including NFKB1, RELA, and STAT1, as upstream regulators of the three biomarkers, highlighting their control by central inflammatory axes (Fig. 6A). Drug-gene interaction analysis identified potential therapeutic agents; notably, Avoralstat and BTI-410 were predicted to target KNG1, while REG3A showed interactions with Sodium Chloride, providing preliminary leads for targeted intervention (Fig. 6B).

## Discussion

### Summary and significance

We have tackled the challenge of early NEC diagnosis through the development of a diagnostic nomogram. This nomogram is built upon a robust three-gene inflammatory signature comprising REG3A, CXCL9, and KNG1. Leveraging high-throughput transcriptomics and employing dual machine learning techniques (LASSO and Boruta), we effectively filtered out noise from the complex high-dimensional data. Consequently, we successfully pinpointed biomarkers exhibiting a high diagnostic accuracy, as evidenced by an impressive AUC value of 0.956. Unlike nonspecific clinical indicators, these biomarkers are intricately linked to the fundamental pathophysiological processes underlying NEC. Specifically, they are associated with muscle damage, adaptive immune activation, and vascular dysregulation, shedding light on the core molecular mechanisms at play in this condition. Thus, our work not only provides a quantitative tool for predicting risk stratification but also offers novel insights into the molecular dynamics of NEC.

### Pathophysiological insights

The biomarkers discovered in this study offer a comprehensive insight into the progression of necrotizing enterocolitis (NEC), delineating distinct yet interconnected pathological stages: mucosal barrier function, adaptive immune response, and vascular dysregulation. One key biomarker is Regenerating islet-derived protein 3 alpha (REG3A), which plays a crucial role in maintaining mucosal barrier integrity. REG3A, a C-type lectin, is known for its potent antimicrobial characteristics and is primarily secreted by Paneth cells and enterocytes [8], REG3A maintains separation of host epithelium and luminal microbiota. In NEC, the breaking of the immature intestinal barrier is a key initiating event [9], the dramatic upregulation of REG3A in our study likely is likely a compensatory host defense to prevent mucosal invasion and restore symbiosis [10]. The marked increase of REG3A is direct evidence of a robust, possibly excessive defense by intestinal epithelium to invasion.Reg3A is thus a sensitive molecular indicator of severity of epithelal stress and extent of barrier compromise.

REG3A is the first barrier defense, CXCL9 is the next step in the inflammatory loop.From initial mucosal injury to transmural necrosis, the immune response is uncontrolled.CXCL9, a chemokine induced by IFN-, acts as a specific chemoattractant for CXCR3 + immune cells (Th1 cells and NK cells).High expression indicates a shift towards a Th1 polarized adaptive immune response in NEC tissues. This recruitment of cytotoxic cells increases local inflammation and leads to intestinal tissue damage [11]. Our results show how adaptive immunity plays in propagating NEC pathology, suggesting that the CXCL9/CXCR3 axis could be a target for interrupting this loop.

In conclusion, KNG1 shows the role of the vascular dysfunctions in NEC. Perhaps the most interesting finding of this paper is KNG1 is a part of plasma kallikrein-kinin system (KKS). Bradykinin is a vasoactive agent known for vasodilation and vascular collapse/u.Livingly NEC is characterized by intestinal wall edema, capillary leakage and potential pathogenicity for systemic septic shock. Upregulation of KNG1 is a molecular mechanism linking local inflammation to systemic collapse [12]. This position KNG1 is both a diagnostic marker and potentially a target for NEC-associated circulatory failure.

### Methodological strengths and clinical utility

One of the strengths of this paper is the use of a “Dual Machine Learning” screening strategy. Transcripts often suffer from multicollinearity and overfitting. Using the intersection of the results of LASSO (a regularization method) and Boruta (a random forest feature selection method), we ensured that the selected biomarkers are not statistical artifacts but robust features with true predictive power. From clinical point of view, the constructed nomogram was high accurate and accurate. Importantly, Decision Curve Analysis (DCA) showed that using this model provides a net net advantage over the “treat-all” or “treat-none” strategy for a broad range of threshold probabilities. This suggests that the model could clinically help neonatologists distinguish high risk NEC patients from benign feeding intolerance, potentially cutting unnecessary antibiotics or surgery.

### Limitations and future directions

However, these promising results have some limitations: First, we retrospectively used tissue transcriptomics.Tissue markers can help us understand the mechanistic properties but invasive biopsy is not a very common early diagnosis. We need to validate the circulating levels of REG3A, CXCL9, KNG1 in noninvasive samples (e.g., serum, stool or urine) to develop a practical liquid biopsy tool [13, 14]. And the small retrospective datasets may cause accidental data bias and overfitting of the machine learning model, which may affect the stability and universality of the diagnostic signature. Second, our sample size was enough for discovery but validation in larger, multicenter prospective cohorts is necessary to confirm generalisability [15]. Finally, although we predicted possible drug targets (e.g., Avoralstat targeting KNG1), experimental verification using NEC animal models or intestinal organoids is required to determine their therapeutic effectiveness [16]. The functional and mechanistic analysis of REG3A, CXCL9, and KNG1 in NEC pathogenesis is only based on bioinformatics prediction and theoretical speculation, without in vitro and in vivo experimental verification. The relevant mechanistic conclusions are limited and need further basic experimental exploration and confirmation in future studies.The subsequent prospective studies will strictly collect and sort out complete clinical baseline information of patients to avoid the interference of clinical confounding factors and improve the reliability of the research results.

## Conclusion

In summary, we found a specific three-gene signature (REG3A, CXCL9, KNG1) which is helpful to diagnose NEC. These three biomarkers represent the “perfect storm” of NEC pathology: barrier failure, immune overactivation and vascular collapse. This work will serve as a foundation for precision diagnostic tools and targeted treatments for NEC.

## Data Availability

No datasets were generated or analysed during the current study.
